# Histopathological Records of Oral and Maxillofacial Lesions among Pediatric and Adolescent Patients in Sulaimani Governorate

**DOI:** 10.3390/children9020156

**Published:** 2022-01-26

**Authors:** Dena Nadhim Mohammad, Ban Falih Ibraheem, Balkees Taha Garib, Marwa Abdul-Salam Hamied

**Affiliations:** Oral and Maxillofacial Pathology, Oral Diagnosis Department, College of Dentistry, University of Sulaimani, Sulaimani 46001, Iraq; ban.ibraheem@univsul.edu.iq (B.F.I.); balkees.garib@univsul.edu.iq (B.T.G.); marwa.hamied@univsul.edu.iq (M.A.-S.H.)

**Keywords:** pediatric, OMFLs, diagnosis, reactive lesions, oral health

## Abstract

Oral and maxillofacial lesions (OMFLs) in pediatrics differ markedly from their adult counterparts in terms of clinical conduct, pathological behavior, and management. This study aims to determine the frequency of OMFLs among pediatric and adolescent patients and to correlate the demographics information to the site, and histopathological findings. Pathological records of pediatric and adolescent patients were retrieved from three major pathological centers in Sulaimani city of Iraq. Demographic information, surgical procedure, anatomical sites, and histopathological diagnosis were recorded. Furthermore, The World Health Organization (WHO) International Statistical Classification of Diseases and Related Health Problems (ICD-10) was used for coding. A Chi-square test was used to find the relation between different variables, and a *p*-value < 0.05 was considered statistically significant. This study archived 309 (13.3%) out of 2319 pediatric and adolescent patients, with a mean age of 11.04 ± 4.62. Females were more commonly detected (52.8%). The most frequently diagnosed lesions were salivary gland diseases (20.7%), followed by reactive hyperplastic connective tissue (18.4%). A significant relation was found between age groups and diagnostic categories (*p* = 0.001). The lips were the most commonly detected sites (20.7%). Mucocele was the most frequently seen non-neoplastic lesions (19.4%), followed by pyogenic granuloma (8.7%). Neoplastic lesions revealed predominant hemangioma (3.2%), followed by peripheral ossifying fibroma (1.9%). Traumatic and or reactive lesions were the most commonly reported lesions. Malignant neoplasms can be identified. The current study enabled systematic data recording of pediatric and adolescent patients, encouraging the importance of the oral healthcare system in identifying and managing the problem early in this critical age in this region.

## 1. Introduction

Pediatric oral and maxillofacial lesions (OMFLs) differ markedly from their adult counterparts in terms of clinical conduct, pathological behavior, and management, with the potential of severely impacting children’s growth and development and thus raising clinical and histopathological concerns [1,2].

The wide anatomical variations of the head and neck region can be responsible for the variety of pathological lesions, hence the broadening of the differential diagnosis and the confounding of the definitive one [3]. Therefore, a thorough examination of the area, and knowledge of various affected age groups with a clear understanding of anatomy and embryology should be sought in order to facilitate reaching a final diagnosis [4].

Pediatric OMFLs can be sorted as congenital, inflammatory, and neoplastic. However, some can be complicated by infection, thus masking the accurate diagnosis [5]. On that account, inclusive comprehension of the prevalence of lesions among infants, children, and adolescents, with an updated awareness among health professionals and medical staff, is essential for identifying and diagnosing such lesions early [6,7].

According to the literature, thyroglossal duct cysts, branchial clefts, and arch anomalies are the most prevalent congenital anomalies among children [8,9,10], while viral or bacterial infections are considered to be the leading cause of cervical lymphadenopathy in young age groups [11,12]. On the other hand, malignant lesions are deemed uncommon and are spotted later in childhood or in the teenage years; however, lesions should be counted as neoplastic and possibly malignant until confirmed otherwise [5,7,13]. Much research from various countries has presented the prevalence of pediatric OMFLs in terms of epidemiological data [1,2,14,15,16,17,18,19]. However, in Iraq, surveys were limited to two studies concerned about children [20,21]. In contrast, most published studies addressed oral lesions in a wide age range, from childhood to the elderly [22,23,24].

Furthermore, studies of data registration and disease classification equip the world’s health system with updated information. The International Classification of Diseases 10th Edition (ICD-10) has a significant impact by using data gathering, monitoring, and analyzing, providing a standard method to compare outcomes across countries [25]. In other words, shared information gathered from different areas at different times would result in universal collaboration leading to a more compatible and systemic gathering of health inputs [26].

This retrospective study seeks to determine the frequency distribution of OMFLs among pediatric and adolescent patients (0–18 years) admitted to three different medical centers in the governorate of Sulaimani/Iraq over the course of 12 years and correlate the demographics information (age and sex), and histopathological findings. Having such data can facilitate arranging probabilities of specific lesions more than others and provide the practitioners with updated information on the prevalence of such lesions in terms of age and sex distribution.

## 2. Materials and Methods

### 2.1. Sample of the Study

The data for this study were collected from the archive of three of the most recognizable pathological referral centers in the city (The pathological departments of the College of Dentistry/Sulaimani University, Shahid Saifaldeen Medical Center, and Shorsh pathological Center). Ethical approval was granted from the Local Ethical and Scientific Committee in the College of Dentistry/University of Sulaimani/Iraq (code no. 34/21) to conduct a twelve-year (2008–2019) retrospective study of OMFLs observed in pediatrics and adolescents’ patients.

The registered pathological records of pediatric and adolescent patients were retrieved. Medical files with complete demographic information (age and sex), type of biopsy, anatomical site and histopathological diagnosis were assembled. Pathological records were categorized into different diagnostic groups. They were then additionally assorted into neoplastic (benign and malignant) and non-neoplastic lesions. The WHO International Statistical Classification of Diseases and Related Health Problems (ICD-10) was also used [26]. Patients from 0–18 years old with surgically removed OMFLs were enrolled in this study and were categorized into four age groups (five years and under, 6–10 years, 11–15 years, and16–18 years), while older patients and those with intra-orbital and thyroid lesions were excluded. Based on these criteria, the database included 309 patients.

### 2.2. Statistical Analysis

The data was statistically assessed using IBM SPSS Statistics version 25.0 software for windows and evaluated by Chi-square test and the relationship between the tested variables were detected by a Pearson Chi-Square Test. A *p*-value < 0.05 was considered statistically significant.

## 3. Results

Throughout the study time, 2319 cases were reported, of which 309 (13.3%) were pediatric and adolescent patients. The average age of patients ranged from near birth 0–18 years old, with a mean age of 11.04 ± 4.62 (male 11.18 ± 4.59, female 10.9 ± 4.6). The majority of cases were reported in the third age group (*N* = 135, 43.7%), followed by the second age group (*N* = 67, 21.7%). The recorded sample revealed a slight predominance of female patients (*N* = 164, 53.1%) over male patients (*N* = 144, 46.9%) with a M:F ratio of 1:1.1 (Table 1). However, the information of the gender and type of biopsy in two separate cases was missing.

The majority of specimens were obtained by excisional biopsy (*N* = 263, 86%), whereas fewer surgical samples were acquired by incisional and incisional/excisional biopsies (*N* = 36, 11.8%. *N* = 9, 2.2% respectively). Furthermore, the type of biopsy was strongly associated with diagnostic categories (*p* = 0.000) (Table 1).

The most common diagnostic categories were salivary gland diseases (*N* = 64, 20.7%), followed by reactive hyperplasia of the connective tissues (*N* = 57, 18.4%), and cystic lesions (*N* = 38,12.4%). The diagnostic categories of lesions appeared to be highly affected by the patients’ age (*p* = 0.001). Meanwhile, gender showed no influence on the pathological diagnosis (*p* = 0.614). The distribution of lesions among various diagnostic categories according to demographic data and type of biopsy was shown in (Table 1).

**Figure 1:**
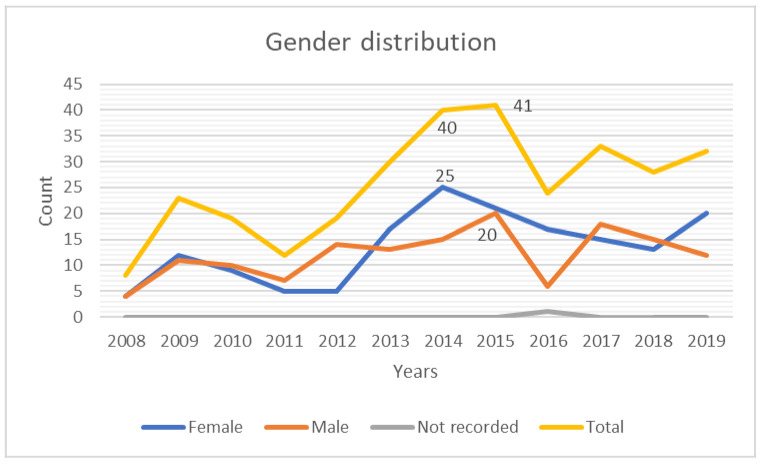
Sex distribution in years 2008–2019.

The peak of reported cases occurred in 2015 (*N* = 41, 13.3%), followed by 2014 (*N* = 40, 12.9%); both years showed female predilection in reporting cases (*N* = 21, 25 respectively), while minimal cases were recorded in 2008 (*N* = 4, 2.6%). The remaining data scattering among female and male patients between 2008–2019 was illustrated in Figure 1.

**Figure 2:**
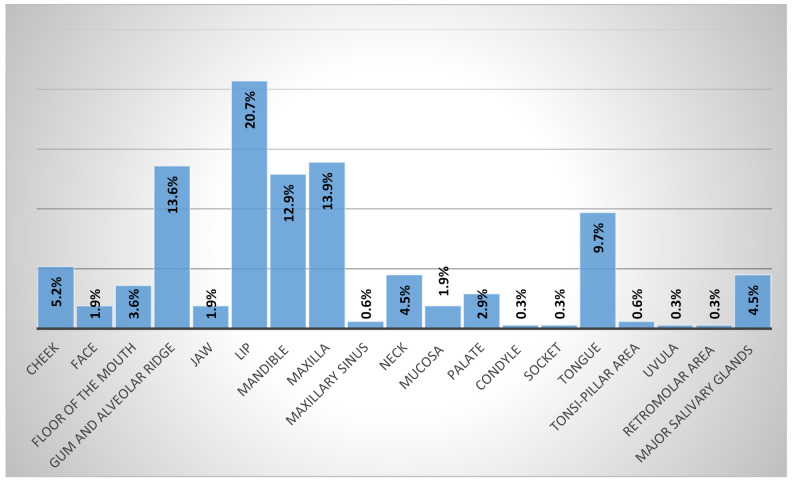
The frequency of oral and maxillofacial sites.

Regarding the site distribution of the sample, the lips were the most affected sites (*N* = 64, 20.7%), followed by the maxilla (*N* = 43, 13.9%) and the gum and alveolar ridge (*N* = 42, 13.6%). Finally, significant relations were detected between the site and histopathological diagnosis and age groups (*p* = 0.000, 0.001), respectively (Figure 2).

A significant relation was found between the site of lesions and the diagnosis (*p* = 0.000), and the site of lesions with the age of the patients (*p* = 0.001).

The diagnosed categories were furtherly classified into neoplastic and non-neoplastic lesions. In non-neoplastic lesions, mucocele was the most prevalent disease (*N* = 60, 19.4%), followed by pyogenic granuloma (*N* = 27, 8.7%)., and periapical cyst (*N* = 21, 6.8%). Meanwhile, among non-inflammatory cystic lesions, kerato-odontogenic cysts (KOC) (*N* = 11, 3.5%), dentigerous cysts (*N* = 10, 3.2%), and developmental cysts (*N* = 7, 2.2%) were most commonly seen. On the other hand, central giant cell granuloma (CGCG) was the most frequent bone lesion (*N* = 7, 2.2%). However, it was less prominent than its soft tissue counterpart (peripheral giant cell granuloma PGCG) (*N* = 16, 5.2%) (Table 2).

Connective tissue tumors (*N* = 36, 11.6%) outnumbered the remaining neoplastic lesions, in which hemangioma was the most prevalent (*N* = 10, 3.2%), followed by peripheral ossifying fibroma (*N* = 6, 1.9%), having a higher frequency than ossifying fibroma of the bone (*N* = 2, 0.6%). Moreover, salivary gland tumor (*N* = 7, 2.3%) showed slight contiguity between benign (pleomorphic adenoma 1.3%, *N* = 4) and malignant lesions (mucoepidermoid carcinoma 1%, *N* = 3). Also, odontogenic tumors showed proximity in number, with Ameloblastic fibroma being the most prevalent (*N* = 3, 1%) (Table 3). The number, age and sex distribution of malignant lesions observed in this investigation were shown in Table 4.

## 4. Discussion

Pediatrics OMFLs vary widely from those of adults; this may be due to the effect of their continuous growth and development with high physical activity. In addition, the prevalence of these lesions among different populations may be influenced by racial genetics, environmental variables, and population lifestyle [27]. Despite the significant impact of OMFLs on the overall health and the WHO recommendations for lesions’ epidemiological assessment, the Iraqi literature provided limited inputs regarding this issue. Meanwhile, most local investigations focused on the prevalence of dental caries [28,29,30]. This study is the first to analyze OMFLs in both pediatrics and adolescents in the Kurdistan region and in Iraq in general.

The total percentage of lesions observed was 13.3%, which was higher than the literature’s prediction [16,17,31]. According to which, pediatric lesions did not surpass 10% of lesions in the general population. However, the increase in the frequency could be attributable to the inclusion of adolescent patients and the various oral health care systems in different parts of the world.

This study revealed the precedence of benign lesions (neoplastic and non-neoplastic), accounting for 97% of OMFLs, a finding that was similar to other two studies in Greek and Thailand [17,32]; meanwhile, malignant lesions were rated at only (3%), being less than those reported in Jordan [31], Colombia [33], and Taiwan [34]; which were 7.2%, 9%, and 5%, respectively, and higher than the frequencies detected by other countries. [17,18,32,35] and a systematic review (1.93%) [36], Despite their rarity, the malignant lesions might mimic benign lesions in the clinical presentation; thus, they should be evaluated in the differential diagnosis of cases with sudden, rapid growth or asymmetric enlargements, as well as ulcerations with unknown local irritants. This disparity in incidences among studies could be attributed to the discrepancy in the time period these studies were held, to the sample size, the geographical region studied, risk factors, or genetic variations.

Despite the considerable number of pertinent studies in the literature, the results can be slightly confusing, owing to variances in the criteria utilized in terms of age range, categorization of lesions, and the various racial and endemic features of the nations involved. In other words, the variable age stratification made it quite challenging to compare the age interval among studies. Some studies in Greece, Saudi Arabia, Iran, and Colombia [17,19,27,33] used a similar age interval (0–18 years), while other research recruited only children [15,16,20,35,37], and pediatric patients [1,18,32,38,39].

The present study reported the highest number of lesions in patients aged 11–15 years old. These findings were supported by a wide range of studies [19,20,33,34,40], while Rwakatema and Chindia 2011 [37] revealed predominant 0–5 aged groups. Duhanuthai et al., 2007 [32] disagreed, noting that most patients were under the age of twelve; this can be explained by the age prevalence of the most commonly reported lesion.

In this analysis, the gender distribution revealed a slight female predisposition. A finding was confirmed by a single-center study in Brazil [35] and another study in Chile [38], whereas some research revealed equal gender inclination [16,17,18,32], and others showed male preference [27,33,37,41]. This study found no gender related to the pathological diagnosis, which agreed with Para Sanabria et al., 2018, Lapthanasupkul et al., 2015, and Lie et al., 2014 [33,40,41]; this may be due to the fact that lesions occurring in children and infants didn’t have gender predilections compared to lesions reported in adults, and the structure of the society in which pediatric and adolescents are still within the household of their parents thus, being highly concerned about their health.

In most studies [18,34,35,38,41], as in this study, mucocele was the most reported lesion. Consequently, the lip was the most prevalent site. This finding agreed with maaita2000, and Zuniga 2013, and could be linked to the traumatizing school environment, as children at this age are more susceptible to accidents due to athletic activities. Furthermore, pyogenic granuloma was the most common connective tissue hyperplasia, along with other studies [17,20,32,35,40]. Meanwhile, Lei et al. 2014 [41] showed fibroma predominance. The high percentages of pyogenic granuloma and peripheral giant cell granuloma might be due to insufficient oral hygiene maintenance in this young age group, as poor oral hygiene and calculus are considered critical etiological factors in the relevance of these lesions.

Moreover, 12.4% of OMFLs were cystic lesions, a finding that was less than the result of Dhanuthai et al. 2007 [32] (35%), but higher than that reviewed by Zuniga et al., 2013 in the pediatric Chilean population (7.4%); however, studies in general showed a range of 10.7% to 17.6% for cystic lesions from OMFLs [16,42]. The odontogenic cysts were the predominant cystic lesions seen in this study, which were 7.4% from OMFLs, coinciding with two other studies’ findings despite the difference in the percentage reported by each (9.9% and 34.8%, respectively) [16,32]. Meanwhile, according to the current study categorization, a periapical cyst showed a percentage of (6.8%), being less than that reported in a local study conducted in Baghdad city of Iraq [20] (9.2%). The differences may be related to the various prevalence of caries in the cities in which the examinations were held.

In the matter of neoplastic lesions, Hemangioma was the most common lesion in this study, as well as in previous studies in Turkey, Jordan, Tanzania, and Germany [16,31,37,43], except for studies in Thailand, Brazil, and Southern Taiwan [32,35,41]. It’s crucial to note that hemangiomas don’t always need to be biopsied. As a result, the incidence of hemangioma may be considerably higher than the number of instances described here.

Most of the aforementioned studies were concerned with documenting the prevalence of OMFLs without exploring the association of different variables. In this investigation, as in two other studies [20,33], it was found that age plays a paramount role in the type of lesions detected; also, a highly significant relation was found between the type of lesions and the site at which they were recorded, as described by Parra Sanabria et al. 2018 (*p* = 0.000) [33]. This may emphasize the value of the ICD-10 for documentation, as it can give physicians and medical personnel a clear image of the necessity of accurate site reporting in generating a differential diagnosis and arriving at a definitive one.

## 5. Conclusions

The findings of this study corroborate and add to previous reports on pediatric oral pathology, which revealed that traumatic and or reactive hyperplastic lesions are the most common reasons for seeking dental help in the young age group. However, despite their rarity, malignant neoplasms can be seen in pediatric pathology. Therefore, correct diagnosis and an excellent therapeutic plan can be ensured by completing all medical records and carefully evaluating prominent clinical characteristics, site documentation, as well as histopathological analysis.

## Figures and Tables

**Table 1:** Distribution of the oral diagnosed lesions in response to age group, sex, and type of biopsy.

Diagnosis Group	Age Group				Sex *			Type of Biopsy ≠		
	0–5	6–10	11–15	16–18	Female	Male	Total	Incision	Excision	Inc/Excision
Bone lesions	1	5	7	0	3	10	13 (4.2%)	2	9	2
Bone tumors	1	1	1	3	4	2	6 (1.9%)	2	3	0
Connective tissue tumors	9	10	10	7	20	16	36 (11.6%)	2	32	2
Cystic lesions	5	9	17	7	18	20	38 (12.4%)	8	30	0
Epithelial lesions	2	1	0	3	3	3	6 (1.9%)	0	6	0
Hematologic neoplasm	2	0	2	0	2	2	4 (1.4%)	0	3	1
Hematoma	0	1	3	0	1	3	4 (1.4%)	0	4	0
Infection	0	0	2	2	3	1	4 (1.3%)	2	2	0
Developmental anomaly	2	1	1	0	4	0	4 (1.3%)	0	4	0
Mucocutaneous diseases	0	1	0	3	3	1	4 (1.3%)	2	2	0
Mucositis/ulceration	2	1	2	1	4	2	6 (1.9%)	1	5	0
Normal	1	0	5	1	1	6	7 (2.3%)	5	2	0
Odontogenic tumors	7	0	1	1	2	7	9 (2.9%)	5	0	4
Periapical lesions	2	1	14	12	18	10	29 *(9.3%)	4	25	0
Periodontal diseases	1	0	0	1	2	0	2 (0.6%)	0	2	0
Reactive hyperplasia of Lymph node	1	0	1	0	1	1	2 (0.6%)	0	2	0
Salivary gland diseases	7	21	33	3	36	28	64 (20.7%)	1	63	0
Salivary gland tumors	0	0	6	1	2	5	7 (2.3%)	0	7	0
Inflammation	1	1	0	0	1	1	2 (0.6%)	0	2	0
Reactive hyperplasia of connective tissue	5	14	28	10	34	23	57 (18.4%)	2	55	0
epithelial tumors	1	0	2	1	1	3	4 (1.3%)	0	4	0
Physical and chemical injury	1	0	0	0	1	0	1 (0.3%)	0	1	0
Total	51 (16.5%)	67 (21.7%)	135 (43.7%)	56 (18.1%)	164 (53.1%)	144 (46.9%)	309 (100%)	36 (11.8%)	263 (86%)	9 (2.2%)

* The sex (*) and the type of biopsy (≠) of two separate cases were not reported (consisting of 0.3% of the total cases), with Pearson Chi-Square Test: A relation was examined between the following.: age and diagnosis groups (*p* = 0.000), sex and diagnosis groups (*p* = 0.614) and type of biopsy and diagnosis groups (*p* = 0.000).

**Table 2:** The distribution of the sample in response to sub-diagnosis of non-neoplastic lesions.

			Sex		Age Group			
Diagnosis Group	Lesions	No. and %	Female	Male	0–5	6–10	11–15	16–18
Infection	Verrucous vulgaris	1 (0.3%)	1	0	0	0	1	0
	Toxoplasmosis	1 (0.3%)	1	0	0	0	1	0
	Actinomycosis	1 (0.3%)	0	1	0	0	0	1
	Granulomatosis	1 (0.3%)	1	0	0	0	0	1
Inflammation	Chronic sinusitis	2 (0.6%)	1	1	1	1	0	0
Bone lesions	Cherubism	1 (0.3%)	1	0	1	0	0	0
	Central giant cell granuloma	7 (2.2%)	1	6	0	2	5	0
	Osteomyelitis	3 (0.9%)	1	2	0	2	1	0
	Fibrous dysplasia	2 (0.6%)	0	2	0	1	1	0
Developmental anomaly	Macroglossia	1 (0.3%)	1	0	1	0	0	0
	Congenital epulis	3 (0.9%)	3	0	1	1	1	0
Mucositis	Non-specific ulcer	6 (1.9%)	4	2	2	1	2	1
Normal tissue	Normal	7 (2.3%)	1	6	1	0	5	1
Mucocutaneous diseases	Pemphigus vulgaris	2 (0.6%)	2	0	0	0	0	2
	Lichen planus	1 (0.3%)	1	0	0	0	0	1
	Lichenoid reaction	1 (0.3%)	0	1	0	1	0	0
Cystic lesions	Kerato-odontogenic cyst	11 (3.5%)	3	8	0	1	7	3
	Aneurysmal bone cysts	4 (1.3%)	4	0	2	0	0	2
	Dentigerous cysts	10 (3.2%)	1	9	0	5	5	0
	Developmental cysts *	7 (2.2%)	5	2	1	2	4	0
	Dermoid-Epidermoid cysts	4 (1.2%)	3	1	2	1	0	1
	Eruption cysts	1 (0.3%)	1	0	0	0	1	0
	Calcifying odontogenic cyst	1 (0.3%	1	0	0	0	0	1
Periapical lesions	Granuloma	6 (1.9%)	4	2	0	0	4	2
	Abscess	2 (0.6%)	2	0	1	1	0	0
	Periapical cyst	21 (6.8%) ≠	12	8	1	0	10	10
Reactive hyperplasia of LN	Reactive hyperplasia of Lymph nodes	2 (0.6%)	1	1	1	0	1	0
Periodontal diseases	Chronic gingivitis	1 (0.3%)	1	0	1	0	0	0
	Gingival fibromatosis	1 (0.3%)	1	0	0	0	0	1
Salivary gland diseases	Mucocele	60 (19.4%)	35	25	7	20	30	3
	Chronic sialadenitis	2 (0.6%)	0	2	0	0	2	0
	Benign lymphoepithelial lesions	1 (0.3%)	1	0	0	0	1	0
	Acute sialadenitis	1 (0.3%)	0	1	0	1	0	0
Reactive hyperplasia of connective tissue	Pyogenic granuloma	27 (8.7%)	18	9	2	7	13	5
	Peripheral giant cell granuloma	16 (5.2%)	8	8	2	4	8	2
	Fibro-epithelial polyp	14 (4.6%)	8	6	1	3	7	3
Physical and chemical injuries	Eosinophilic granuloma	1 (0.3%)	1	0	1	0	0	0
Hematoma	Hematoma	4 (1.3%)	1	3	0	1	3	0
Epithelial lesions	Hyperkeratosis	2 (0.6%)	1	1	0	1	0	1
	Hyperplasia	2 (0.6%)	0	2	2	0	0	0
	Intradermal nevus	1 (0.3%)	1	0	0	0	0	1
	Dysplasia	1 (0.3%)	1	0	0	0	0	1
			Total = 243 (78.6%)					

* Developmental cysts included (5 thyroglossal duct cyst, 1 branchial cleft cyst, 1 inflamed cyst). ≠ sex of one periapical cyst lesion was not reported.

**Table 3:** The distribution of the sample in response to sub-diagnosis of neoplastic lesions.

			Sex		Age Group			
Diagnosis Group	Lesions	No. and %	Female	Male	0–5	6–10	11–15	16–18
Bone tumor	Ossifying fibroma	2 (0.6%)	1	1	1	0	0	1
	Osteochondroma	1 (0.3%)	1	0	0	0	0	1
	Osteoma	1 (0.3%)	1	0	0	0	0	1
	Osteosarcoma	1 (0.3%)	1	0	0	1	0	0
	Ewing’s sarcoma	1 (0.3%)	0	1	0	0	1	0
Hematologic neoplasms	Burkitt’s lymphoma	1 (0.3%)	1	0	1	0	0	0
	Lymphoma	1 (0.3%)	0	1	0	0	1	0
	Langerhans cell histiocytosis X	2 (0.6%)	1	1	1	0	1	0
Connective tissue tumors	Hemangiopericytoma	3 (0.9%)	1	2	1	1	1	0
	Nodular fasciitis	3 (0.9%)	2	1	3	0	0	0
	Hemangioma	10 (3.2%)	5	5	2	2	3	3
	Cystic hygroma	2 (0.6%)	2	0	1	0	1	0
	Neural tumor	3 (0.9%)	0	3	1	0	2	0
	Lymphangioma	3 (0.9%)	2	1	0	2	0	1
	Lipoma	1 (0.3%)	1	0	0	1	0	0
	Fibroma	1 (0.3%)	1	0	0	1	0	0
	Peripheral ossifying fibroma	6 (1.9%)	6	0	0	1	3	2
	Lymphangioma-hemangioma	2 (0.6%)	0	2	0	2	0	0
	Rhabdomyosarcoma	2 (0.6%)	0	2	1	0	0	1
Salivary gland tumors	Pleomorphic adenoma	4 (1.3%)	2	2	0	0	3	1
	Mucoepidermoid carcinoma	3 (1%)	0	3	0	0	3	0
Epithelial tumors	Squamous papilloma	3 (1%)	1	2	1	0	1	1
	Melanoma	1 (0.3%)	0	1	0	0	1	0
Odontogenic tumors	Ameloblastic fibroma	3 (1%)	0	3	3	0	0	0
	Ameloblastoma	1 (0.3%)	1	0	1	0	0	0
	Odontoma	2 (0.6%)	0	2	1	0	0	1
	Odontogenic fibroma	1 (0.3%)	0	1	0	0	1	0
	Melanotic neuroectodermal tumor	2 (0.6%)	1	1	2	0	0	0
			Total = 66 (21.4%)					

**Table 4:** The distribution of malignant lesions in terms of age and sex.

Malignancies	Age Group				Sex	
	0–5	6–10	11–15	16–18	Female	Male
Osteosarcoma	0	1	0	0	1	0
Ewing’s sarcoma	0	0	1	0	0	1
Burkitt’s lymphoma	1	0	0	0	1	0
Lymphoma	0	0	1	0	0	1
Melanoma	0	0	1	0	0	1
Mucoepidermoid	0	0	3	0	0	3
Rhabdomyosarcoma	1	0	0	1	0	2
Total 10 (3%)	2 (0.6%)	1 (0.3%)	6 (1.8%)	1 (0.3%)	2 (0.6%)	8 (2.4%)

## Data Availability

Data will be available on request through this mail dena.mohammad@univsul.edu.iq.
